# Discounted Deaths: The Eruption of COVID‐19 in the Geriatric System of the Community of Madrid

**DOI:** 10.1111/maq.12730

**Published:** 2022-09-12

**Authors:** Iñaki Rubio‐Mengual, Álvaro Villar Baile

**Affiliations:** ^1^ Department of Sociology and Social Work University of the Basque Country Leioa Vizcaya Spain; ^2^ Department of Sociology and Social Work University of the Basque Country Leioa Vizcaya Spain

## Abstract

In this article, we explore a new category of analysis that we have called “discounted deaths,” with which we seek to examine forms of dying occurring outside the scope of the triple meaning of the term “to count”—i.e., deaths that did not count, deaths that were not counted, and deaths for which there was no account. To do this, we look at the empirical case of elderly people who died in Madrid's nursing homes during the first wave of the pandemic, between March and May 2020. Compared to other affected groups, theirs were deaths that were deemed tolerable. People who died in nursing homes were first excluded from the assistance mechanisms available under the health emergency and then buried in solitude, away from their loved ones, who were not made aware of their situation until they were in their final moments. [coronavirus, death, account, old age, Madrid]

## Introduction

In 1842, Russian writer Nikolai Gogol published what was to become his most celebrated work: *Dead Souls* (Gogol, 2004). In this novel, divided into two parts, the author chronicles the exploits of a minor member of the nobility called Chichicov, who travels across the Russian steppe recording the deaths of peasants who have succumbed to the spread of diseases, with the aim of securing the rights to the plots of land they farmed. His actions illustrate how even when it is impossible to safeguard the lives of populations affected by epidemics, there is, nonetheless, a need to manage what remains of the dead, taking them out of a state of registry indeterminacy by first bringing them symbolically back to life, restoring their name and history, to then certify their death. Underlying the story is the bitter distortion of the vital status of those bodies, which are neither dead nor alive. Having fallen victim to rural plagues, they were scattered throughout the rough terrain of the Slavic tundra, as they waited to join the ranks of the officially dead once the traveler registered their deaths. The novel is a satire of its own era, throwing into stark relief the incongruities and inconsistencies of a feudal society that was taking its first steps in the development of a modern state that would not be fully consolidated until well into the following century. As we will see in the following pages, this idea of “counting death after death has occurred” resonates more than 150 years later in what happened in some corners of the Spanish health care system during the early months of the COVID‐19 pandemic.

Between February and May 2020, in the initial outbreak, death emerged in Spain's nursing homes as a phenomenon that was both massive and solitary (Amnesty International 2020). Administrative and political decisions allowed the virus to spread through the corridors of these facilities, creating an eerily synchronic dynamic in which most residents fell simultaneously ill and were confined to their rooms. In these circumstances, the virus worsened previously vulnerable situations, taking them to the point of death within days and in strict isolation. What had until then been places where elderly people went to live out their final years turned into manifestly morbid scenarios, filled with people dying suddenly in droves, struck down by something that at the time could not be explained. These were discounted deaths, occurring in situations of abandonment in a context of exceptionality, intensifying pre‐existing dynamics of care/abandonment that could already be observed in conventional care‐giving and record‐keeping (Garcia 2010). Once the most critical moments of the first wave had passed, these losses would seem to have occurred without leaving a trace.

This study is based on fieldwork carried out between April 2020 and July 2021 in and around Spanish nursing homes and hospitals. Given the impossibility of undertaking an ethnography during the first wave, we opted for developing a methodology that combined three sources of data. First, we conducted a total of six interviews with relatives of nursing‐home residents who had died in those geriatric institutions and with health care professionals, both from such institutions and from hospitals. As supporting material for the interviews, we obtained medical records that were made available to us by relatives of the deceased residents. Second, we gained access to the Emergency Military Unit (Unidad Militar de Emergencias, or UME) through an information channel, which gave us with a copy of the Operation Balmis report, an extensive confidential document listing the various procedures and interventions carried out by this military unit. Finally, we conducted a statistical analysis of the records of infections and deaths, provided by the health departments of the different Autonomous Communities.

## Tolerable Deaths, Truncated Mourning

During those months, we witnessed a confusing spectacle in which laws, meetings, papers, epidemiological maps, and statistical projections produced internally, with no media coverage, descended on that reality in the form of ambulances, paramedics, medical partition screens, and ventilators whose distribution and effectiveness would determine the survival of the individuals affected by the disease. When examining whether infected nursing‐home residents in the Community of Madrid were referred to other facilities, we identified three dimensions corresponding to three different analytical moments—before, during, and after—and which allow us to analyze how the pandemic unfolded within these institutions. These dimensions are: the managing of hospital admissions; the distribution of technical resources; and the administration of the dead.

The first proposals for managing hospital admissions did not come from administrative spheres; rather they were put forward by medical associations. As early as March 2020, the Spanish Association of Intensive Care Medicine, Critical Care, and Coronary Units (Sociedad Española de Medicina Intensiva, Crítica y Unidades Coronarias, or SEMICYUC) published an article outlining a protocol that anticipated the hard decisions that awaited Spain's health care system, based on how SARS‐COV‐2 was spreading in other countries. The article foresaw the need to consider the ethical dilemma that would be created by the likely saturation of the available means for combating the virus—primarily, means of detection and prevention, invasive mechanical ventilation, and ICU beds—as hospitalizations increased progressively. This dilemma was posed in terms of catastrophe medicine,^1^ which meant organizing medical assistance by rationing available resources to strike a certain balance. That balance would be guided by two considerations: on the one hand, the patient's maximum possibilities of surviving the virus, which entailed neglecting patients whose condition worsened more quickly; and on the other, the maximum number of years that could be ensured for that patient after hospital discharge, which entailed neglecting patients who suffered from severe pre‐existing illnesses (Rubio et al. 2020).

A few weeks later, we were facing scenarios that heralded a potential collapse of the public health care system. The saturation of available intensive care units led to a high concentration of patients in adjoining facilities, both inside and outside hospitals, where those suffering the most severe effects of the virus began to die. Such facilities included corridors, waiting rooms, lobbies, and makeshift tents. In the Community of Madrid (where we focused our analysis), political planning was implemented—paradoxically enough—through institutional inventive processes endorsed by the central government, which consisted of converting public infrastructures allocated for other purposes into spaces that would absorb the needs that could no longer be met by conventional hospitals nor by the above provisional spaces. During our fieldwork, one nursing home director told us that they had to work “without any structure or precedent, without any decree, without any instructions about what we had to do; intuitively.” A doctor explained that this process was experienced “as if it were a bombardment that swept everything away. An entire physical and functional structure had to be improvised in just a few days.”

The most relevant case, in this sense, was the Institución Ferial de Madrid (IFEMA) hospital, an association headquartered in the northeastern district of Hortaleza, with grounds spanning 200,000 m^2^ and housing 12 pavilions, three of which were used during the health crisis. It is a venue used for various international events held annually, ranging from contemporary art fairs to fashion shows and economic conventions for various industries (Institución Ferial de Madrid 2021). From March 23 through April 2, 2020, a provisional health care complex was set up at that venue with the aim of satisfying the demand that had overwhelmed Madrid's leading hospitals. This temporary hospital contributed 1,300 beds, 16 of them equipped with intensive care resources, half of which were managed by military health care personnel (Munayco et al. 2020). It was called a “field hospital” and its deployment was part of a wider action (known as “Operation Balmis”). It was a series of actions carried out by Spain's armed forces, starting on March 15, 2020, under the command of the Defense Ministry. The general aim of this operation was to provide logistic transportation for social and health care professionals and medical resources, supply military medical personnel, set up field hospitals, remove bodies, and disinfect homes and hospitals. To do this, a strategic reform of the military organizational and territorial structure was undertaken, to allow the armed forces to intervene exceptionally in the prevailing circumstances, which could not be managed with civilian means (Ministry of Defense 2021).

Much of the army personnel worked side by side with civilian health care professionals to address the situation, so that the discipline of the hospital system was combined with military command protocols in a space where the biopolitical maxim “make live and let die” (Foucault 1978) took on a literal quality. This situation recalled contexts that seemed already forgotten in Spain, such as the AIDS pandemic in the 1980s or the Ebola crisis in 2014 (Morales 2021). Thus, it became a priority to keep patients who could not be treated at conventional facilities alive and, in this sense, any available spaces in this temporary shelter were assigned to patients who “needed to be hospitalized, but were less serious and did not have multiple pathologies” (Hernández et al. 2021: 521). In other words, this field hospital was reserved for people who were “savable.” Although it was set up in record time, its very nature evidenced the existence of a “raison d’état” (or national interest) that meant denying the new resources to those individuals who were hardest to cure (Rose et al. 2006).^2^ Certain sectors of the population, which had a low life expectancy due to chronic illnesses or situations of dependency, were left untreated and confined, to avoid further contagion. This responded to an immunological strategy aimed at preserving certain lives to the detriment of others, who needed to be isolated (Esposito 2008). This dynamic especially affected elderly people living in nursing homes in the Community of Madrid.^3^ These facilities applied exclusion criteria, which entailed shutting down hospital referral channels for residents who had contracted the virus. This resulted in people dying without receiving due care, in places that, paradoxically, were meant to guarantee their well‐being. This plan was known as Protocol for Action in Nursing Homes in the Community of Madrid during the Epidemic Period Caused by COVID‐19 (Community of Madrid 2020b).

This protocol brought to the surface latent hospital care logics in Spain, which were now shaped by new guiding principles.^4^ From then on, the severity of the disease—understood as its potential to cause a patient's death—would no longer be the reason that justified medical intervention, nor would it be a determining factor in how far that medical intervention would go to cure the patient. Medical intervention began to be linked to the very *viability of life* (Leblanc 2007), namely, the ability to overcome the disease and continue living for a maximum number of years (Rueda 2020). What mattered was not so much that a patient could be cured, but that the cure be applied to a body that could be projected forward in time. So, someone who was young, previously healthy, and with a high life expectancy, was considered to have a *full* life, and that assessment acted as a supporting factor that ensured that person's continuity in the event of exposure to the virus. Elderly people, in contrast, were denied the means that could have allowed them to continue living (del Pino et al. 2020), since their bodies were now seen as bodies that were already sick (Groisman 2002), especially if those people were institutionalized. Their characterization as decaying life, subject to processes of deterioration that manifest as chronic, placed them at a disadvantage when it came to accessing the necessary life support systems. Thus, chronic illness became a factor that justified the decision not to treat a deadly disease, furthering the view that the lives of the chronically ill or elderly were secondary in terms of protection. This part of the argument vividly echoes the image that Cazdyn (2012) paints regarding the already dead, a form of contemporary subjectivity that represents the death of a body that is not yet dead, a body that has been abandoned in a no‐man's‐land between an impossible life and an inevitable death.

Certain categories were created to introduce the distinction between projectable and non‐projectable bodies. These categories served to guide institutional action when it came to deciding who to treat first and who to relegate to lesser forms of care, in what can be understood as a biological metric of sorts. The protocol established by the Community of Madrid for geriatric institutions distinguishes between mild, moderate, and severe COVID‐19 patients. Under each of these categories, the health condition of the patient prior to contracting the virus is then considered (Annexes I and V). Nursing home residents were tested only “exceptionally and depending on hospital availability” (Community of Madrid 2020b: 10), and hospital referrals were ruled out either because the patient had a mild case—potentially treatable in the nursing home—or because their condition worsened and it was considered a futile effort to provide the necessary means to combat the disease. In this way, the categories employed separated elderly people from the rest of the population, with the former left where they were and the latter treated in hospitals. This form of selection, far from being new, is reminiscent of an old ethical distinction that reemerges with force in a strange context. That distinction entailed rehabilitating valid bodies and separating invalid bodies, with the latter understood as those whose damage cannot be repaired (Castel 1995).

A second dimension to be considered when thinking about the consideration of life as a value in itself is the distribution of technical resources aimed at preserving life. These resources, which were completely overwhelmed by the circumstances, include all the instruments and means used to protect staff, care for the sick, and run basic operations in a health care system.

Given that, at that time, the virus acted as an invisible evil—difficult to detect and with unpredictable consequences—the availability of personal protective equipment (PPE) was an issue that was becoming increasingly important. Sterilized gowns, latex gloves, FFP2 masks, and transparent face shields made up the soft armor that separated functional bodies from those who, clad in ordinary clothes, lay in their own beds, biologically exposed to the virus and to intervention (Oyarzun 2020). During the first working days of the pandemic crisis in Madrid's nursing homes, the shortage of this type of product eliminated the only known preventive barrier, thus putting both patients and caregivers at risk.

Another major instrument, whose availability was decisive in determining the last possibility of survival for those facing the direst effects of the virus, was the ventilator. With its ability to provide oxygen to damaged lungs, this machine gives the body precious time to allow the immune system to respond to the threat. The number of units available determined the number of seriously ill patients who could be treated, placing an insurmountable limit that would force a reconsideration of the hospital logic that had operated until then, as noted above. The ventilator became at the same time a technical threshold between the life and death of a critical patient, and a *rara avis* or an exceptional resource that determined the decisions regarding who would receive medical care and who would not. We focus on this because it vividly illustrates how biomedical technology—and its distribution—can constitute an intermediate link between large social structures and small ordinary plights. It is a device connecting the biological to the social, turning the act of breathing—an unconscious physiological act—into something than can be subject to political decisions, to the point of defining the possibility of life at its limit (Salomon 2021).

Finally, a third dimension to be considered regarding the importance of life in the case under analysis is the administration of the dead. As we explained above, the process for allocating treatment for serious cases within Madrid's health care system involved considering the overall state of the sick person's body and their life expectancy before COVID to establish treatment priorities accordingly. This, in turn, entailed excluding the elderly, causing a sharp increase in the number of deaths in nursing homes. That increase had to be managed. After the dead were picked up and removed from the homes, they went on to occupy another peculiar space set up during this time: the Palacio de Hielo (or Ice Palace). It is a shopping complex, also located in Madrid's Hortaleza district. The mall has several levels and houses various brand name stores and a food court. There is also an Olympic size ice skating rink, measuring 1,800 m^2^, which is part of the complex and gives it its name (Palacio de Hielo 2020).

This was part of the institutional inventive efforts aimed at alleviating collapsed situations in the health care system caused by the pandemic. In this case, it was a large improvised morgue set up on an ice skating rink to preserve the bodies that were piling up as more and more people died from the virus. This measure was meant to decongest mortuaries in the city's hospitals, nursing homes, and funeral parlors. From March 23 to April 22, 2020, five long carpets were laid across the frozen surface of the rink to accommodate the remains of 1,146 deceased individuals.^5^ During the first wave of the pandemic, this place saw intense daily activity from army personnel and firefighters bringing in the bodies of many of the elderly people who died in nursing homes. As bodies were transferred to the rink, the worst hit nursing homes were gradually emptied out. The relatives of the deceased residents were notified that their loved ones were no longer there only after the bodies had been removed.

Thus, the isolation these individuals had been subjected to in life did not end with their death. As if they were toxic waste, their bodies were sealed before they were hurriedly taken out of the nursing homes. They were first put in airtight coffins, then stacked in the back of a truck that transported them to the temporary morgue, and, finally, placed on the ground in rows of coffins that ran from wall to wall of the rink. The process of notifying relatives was not only delayed, it was also done in a disorganized manner. At the morgue, the bodies waited isolated in their coffins for several days. They were laid out side by side in rows, devoid of any symbolic or ritual markings to identify them, and no one from outside the facilities was allowed to access them. Sometime later, they were taken to community mausoleums in cemeteries indicated by their relatives, in a funeral procession that was merely procedural, attended by a handful of their loved ones, following the strict protocols devised specifically for those circumstances, and with no liturgical elements other than the presence of a priest as officiating witness.

In his book *The Dominion of the Dead*, Robert Pogue (2003) describes burials as the rites that allow us to incorporate our dead into the sphere of the everyday, that is, that which presents the living as a continuation of the loss, with the body being the personification of it all. In the scenario we discuss here, death seems to have occurred with no affective background—i.e., without a physical place, outside of time, and, above all, without witnesses participating in a shared mourning. In this regard, one of our interviewees told us that she and her sister were lucky, because they managed to keep their mother's body in the morgue for only two days, and they did not have to travel hundreds of kilometers as a friend of hers did. However, she recalls with much sorrow that they were not able to identify her mother's body before it was cremated. “We couldn't see her; we only saw the coffin being taken to the cremation chamber.”

Ultimately, the Spanish state's characterization of geriatric institutions as spaces for the protection of vulnerable lives, where caregiving is provided for the elderly and for dependent people, was shattered by the proliferation of harsh terms, such as “neglect” (Doctors Without Borders 2020) and “abandonment” (Amnesty International 2020) used to described the new practices. This furthered the ethical breakdown of such institutions, a breakdown that was circumstantial, brought on by emergency, critical, and temporary measures devised to prevent the general collapse of the social and health care systems. But it also had profound effects on the population of nursing homes, either because the measures adopted did not allow for the protection of these individuals when their lives were at risk, or because they failed to protect them and acted in such a way that exacerbated their vulnerability. The two were sometimes combined, resulting in difficult ethical issues. Drawing on Lawrence Cohen (2020), we can describe Madrid's nursing homes at that time as paradoxical spaces where care and neglect were intertwined to the point of being confused. The same doors that were once opened to welcome those who sought to ensure their own well‐being in the late stages of life were now being shut resoundingly. A hermetic environment was created to keep the residents firmly inside. It became a place they would only leave on a portable stretcher, covered with a dark sheet, and carried out the back door, which during those months saw a lot of activity. In that setting, death sped up, and the state of emergency camouflaged the significance of what happened. In the following section, we address what lies behind these deaths that do not matter, examining how the deaths that occurred in these institutions were recorded and how there is no trace of them in the accounts of the pandemic.

## Numberless Dead and Hidden Populations

Generally thought of as powerful methods for representing the “positive states of the population,” statistical censuses are not so much that as they are tools for the production of data based on reality, even if they may not always match that reality exactly. Their purpose is to translate existing facts into measurable and comparable objects, but that aim is underpinned by a whole process of selection, valorization, and codification, so that it would seem that statistics are a method focused more on their utility for governing and ordering than on their capacity for objective representation (Desrosières 2013). As of the late 19th century, the colonizing process introduced a new approach with a different aim: that of reflecting all of reality (Anderson 1993) so that nothing is left without classifying. In that way, any uncertain, scattered, and confused territories and populations that the metropolis did not know how to bring under its rule are put into categories. This has led to the creation of unexpected subjectivities, which do not fit into official accounts and which give rise to alternative ways of being that are out of step with the dominant rationality, either because they never belonged to it (Stevenson 2014) or because they introduce new sensibilities (Jain 2013).

Besides other major contemporary epidemiological events, such as the AIDS pandemic, when a certain invisibility in the registry could also be observed (Biehl 2005), this statistical vocation has remained practically unchanged. It was one of the leading elements that made it possible for the Spanish government to render the epidemiological progression of the pandemic intelligible and enabled it to make social diagnoses and organize responses. It did so through what it called *Actualizaciones* (updates), issued by the Ministry of Health and published daily. At first, these updates contained little information and were not very detailed, but as the situation evolved, they expanded their scope and developed two formal representation models. The first model was the epidemiological map, which was the preferred form of understanding how the virus spread, where it spread to, and the ways through which it accessed the places it spread to. This was a first step toward identifying viral flows and controlling morbidity. The second model was the mortality census, which made it possible to count the deaths and which became an indicator that aimed to quantify the dimensions of the tragedy. In short, while these two models were intended to describe the reality of the pandemic, they also ended up being dependent on the tools used to build the data they contained: the diagnostic tests.

The widespread shortage of diagnostic tests, their skewed distribution in favor of hospitals, and their probabilistic results made it difficult to record new cases in nursing homes during the first months of the pandemic, resulting in complete uncertainty. There was no information on how many people in these institutions were infected, nor was it possible to establish a direct causality between deaths and the virus (Doctors Without Borders 2020). This took on such relevance that on March 23, two orders were issued that exclusively regulated the recording of cases and deaths in these facilities. Under these regulations, nursing homes had to provide that information twice a week through an administrative chain of reporting that began with the facilities and ended with the central government, but which also included hospitals and the Autonomous Communities, the political and administrative division with the aim of guaranteeing limited autonomy of the nationalities and regions that make up Spain.^6^

In this way, a “two‐speed” accounting system was formalized: a first‐order account or record that was general and verifiable, in which any case could be included; and a second‐order account that was irregular and imprecise. This second‐order account was meant to include the cases that could not be classified in the first account because the mechanisms of detection and recording were not working properly in the places where those cases occurred, and thus could contaminate the general record. Therefore, the tallying of deaths in the geriatric institutions of the Community of Madrid left out numerous cases of people who died from COVID‐19 and who are not reflected in official statistics.^7^ One administrative worker of a nursing home referred to these as cases as remaining “in no‐man's‐land.” A gap of uncounted deaths was generated (Pécoud 2018). This gap cannot be measured despite the efforts of non‐governmental organizations such as Doctors Without Borders, but it can be examined by considering two limitations that affected census taking.

The first limitation can be described using the concept of lost data, which is a common concept in statistical language. What characterizes lost data is that while such data are recorded, they cannot be treated with the consistency of validated data, so that they are featured separately from the rest in an “aside” that cannot be processed. This notion is interesting because a dubious category was applied in the recording of deaths in the Community of Madrid, namely the “patients who died with symptoms compatible with COVID‐19” category, containing all undiagnosed cases. As of April 1, 2021 (a year later), 76% of the recorded COVID‐19‐related deaths had been classified as “deaths with compatible symptomatology.” A total of 4,775 deaths fell under an indeterminate status. They were included among the deaths that were counted, but without the certainty of a diagnosis, which would have allowed these patients to be treated while they were still alive. That lack of diagnosis also meant that once dead, they could not be considered categorically within the figures that serve to give an account of the tragedy. They were recorded as invalidated data, which with the passage of time and with the bodies already buried, can no longer be contrasted or filtered.

The second limitation is more complex, as it consists of the existence of data that slip between the cracks at different points along the channels of detection, recording, and communication so that they never reach the statistics that illustrate the phenomenon. This is what we call detached data. To illustrate this, we draw on material gathered in our fieldwork in the summer of 2020, at which time we conducted an interview with a woman who had lost her mother in a nursing home in the Community of Madrid. Before telling us about how difficult it was for her to mourn the loss of her mother, she told us how her mother, like other elderly people, became ill during an outbreak that occurred in April. The difficulties in providing treatment for her at that time were recorded in a parallel account: her medical history. This record shows that it was not until five days after her first symptoms appeared that she was tested to confirm the presence of the virus in her body. After her condition worsened, she was taken to the hospital, where she remained in the emergency room without access to a ventilator and without the possibility of being transferred to the ICU.

**Figure 1:**
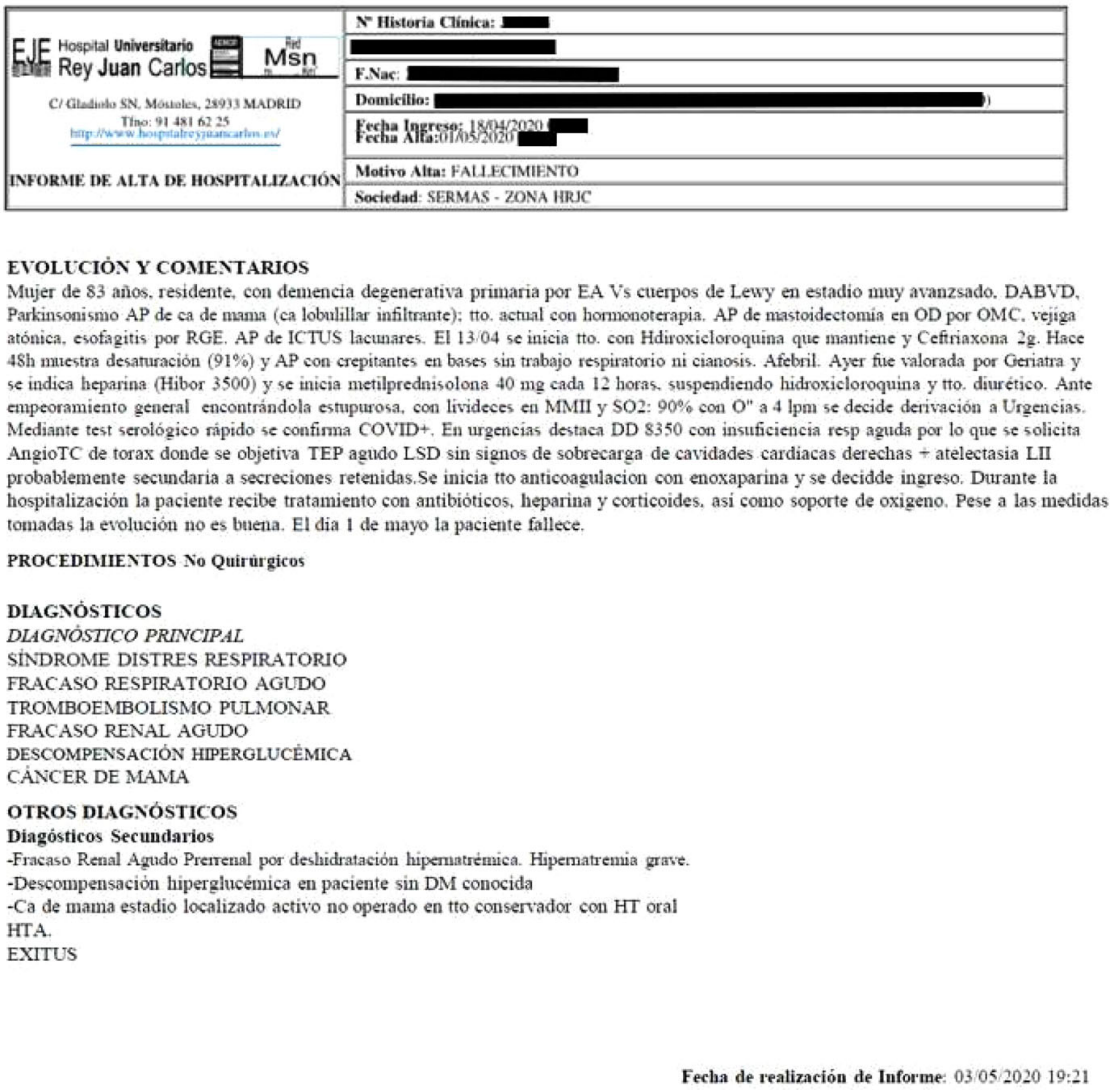
Clinical history of an 83‐year‐old patient residing in a geriatric institution in the Community of Madrid, obtained during fieldwork conducted as part of the research. (Content obtained by the authors) [This figure appears in color in the online issue]

The woman died on May 1. Her daughter, perplexed, showed us her medical history, noting how all mention of COVID‐19 had been removed and replaced with the symptoms resulting from the virus: respiratory distress syndrome, acute respiratory failure, and pulmonary thromboembolism “No assessment is given. I've asked the doctor at the nursing home and she says that the same thing happened with other cases there of people who died from COVID, but for some reason the virus is not mentioned,” she told us. What at first glance appears to be the common progression of the disease as it worsens, conceals an error that prevents the woman's death from being included in the mortality census. The diagnosis misplaced in the offices of the institution leads to the deceased person being erased from the accounts in which she should have been included, making her invisible in the eyes of the state (see Figure 1) (Biehl 2005).

We could say that taken together, these cases form hidden populations (Heckathorn 1997), which have fallen through the cracks of the accounting process that recorded the social effects of the pandemic. They are hidden as a result of an accumulation of errors and accidents in the detection and recording of deaths of elderly people in nursing homes, which, individually, do not amount to much, but, when aggregated, configure a statistical landscape full of leaks and imprecise data.

## Dying without an Account in State Facilities

The usual daily routine of family visits and activities carried out by volunteers gave way to a dulled environment, shaped by social distancing measures that turned the former co‐residents into a mixed group of dead and survivors: mute subjects whose lives were reduced to the “bare minimum of their biological continuity” (Giorgi 2014: 253). A particular background was thus formed, where loss was dissociated from the account. These cases could be said to fall within the sphere of forms of contemporary vulnerability, in what Judith Butler (2015: 196) refers to as “lives [that]do not matter.” What happened in Madrid's nursing homes is what we call here “death without an account,” a process in two acts.

(I) First, there was a paralysis of the institutional setting where the death took place. The nursing home, as a place specialized in managing the last stages of life, was one of the modern spaces where the representations of dying outside the context of the home were established (Ariès 2011). While not a hospital, it is a place where caregiving under the structure of kinship is subordinated to health care practices and knowledge (Donzelot 2005), sometimes resulting in situations in which the residents become detached from their families and these, in turn, are no longer the social locus where the resident's existence as individuals unfolds (Elias 2010). The COVID‐19 crisis led to these people's deaths being perceived as something that occurred offstage—outside of space and time and without an audience that could effectively bear witness to what happened. If we view the situation in terms of the concept of tragedy predominant in Western culture, which is always linked to the eruption of a major event beyond human control (Morris 1996), the first people who died in Madrid's nursing homes were deprived of a role in the drama. They were not heroes, as health care workers were; nor were they victims, as patients treated in hospitals were; and neither were they a combination of the two, that is, martyrs, who were represented by health care workers who died in the line of duty. They were merely casualties that occurred inside an institution that produced deaths devoid of any expression and that were, therefore, excluded from this official account (Butler 2015).

(II) Second, these situations took place out of sight. What happened during and after the above events did so behind closed doors, doors that were at all times impervious to any external controls that could be exercised by groups such as humanitarian organizations, associations of relatives, or personnel from other facilities. By these, we mean agents from outside the sphere of health care practice who could have, perhaps, approached the process of loss compassionately—acknowledging those who were dying as subjects of suffering and not just as carriers of the virus (Kellehear 2005). In any case, the perception of COVID‐19 as an uncontrolled and urgent social problem—sickness—precluded approaches that would take into consideration its experiential dimension—as illness—(Frankenberg 1993), and the overall result was an institutional silence that tried to avoid the most difficult situations. Outside these facilities, the state—in this case, its regional bodies—appropriated the communicative capacity of those affected (Das 1995). It lumped together the suffering experienced in the nursing homes with the state of general commotion and avoided having to give concrete explanations of what went on in such dismal and specific contexts.

At the time of writing this, exactly one year has passed since the first wave was finally contained, more or less ending the controversy over nursing home deaths that had begun a few months earlier in some of the country's media. Since then, despite some new outbreaks and an occasional mention in public debates, the situation appears to have been brought under control without any major incidents. Addressing the issue today entails approaching an empirically scorched field that appears to have been subsequently repopulated, sweeping the irregularities under a smooth surface. Further analysis will entail sorting through the gaps in the documentation currently made available by the institutions, in search of stray data that can provide concrete signs of a dubious category, which will allow us to examine the intricacies of the situation (Boltanski 2014).

## Conclusion: Discounted Deaths as an Analytical Category

In the absence of conceptual tools to name them, we speak of “discounted deaths,” deaths that simply “happened” in a vacuum, unmoored from the meanings provided by the social sciences (Gatti 2005).

The situation of the elderly residents of the nursing homes of the Community of Madrid during the initial months of the pandemic gave rise to deaths that were assumed inevitable by places that were meant to help assist in death (they were deaths that did not count), which slipped through the cracks of the apparatuses specialized in managing the state—i.e., statistics—(deaths that went uncounted), and that lacked a narrative that would make it possible to explain them (deaths without an account). These three processes were the result of two opposing logics: humanitarian reason and raison d’état. With this, we refer to the tension between the concept of life as a supreme value that must be preserved in all its forms (Fassin 2018) and the need to maintain the functions of a saturated state that safeguarded those who were biologically more advantaged, at the cost of letting those who had less future ahead of them go (Rose et al. 2006). In other words, the general health care system, overwhelmed by the circumstances, was able to resist at the expense of allowing the breakdown of a nursing home network that lacked adequate means to care for those who wasted away in its facilities. In that period, the nursing homes were truly bleak contexts in which a “planned omission” of assistance took place. They did not have the necessary means to keep the patients most affected by the virus stable, so they operated instead as an abandonment device (Biehl and Eskerod 2013), generating forms of being that were pierced by suffering and bordered on the unlivable (Butler 2015).

These were lives abandoned to their fate, lives that were lost in a context of structural precariousness and permanent undocumented status (Gatti 2020) and that, once the cloak of exception was lifted, seem to have disappeared suddenly and for good. What was posed in terms of a tragedy and seemed to have strained the geriatric system and the state's duty of protection toward vulnerable populations, today appears to have evaporated without any trace other than what we have tried to gather here, as the diligent Chichicov did in the pages of *Dead Souls*. In the future, this experience should be taken into consideration to rethink situations in which death is discounted, both in the social sciences and in the fields of medical and social intervention.

## Funding information

Ministerio de Ciencia, Innovación y Universidades MICINN PID2020‐113183GB‐I00

## Notes

1 Words from this term's semantic field shaped the way we defined a new situation that intensified the dynamics already present in hospitals. These terms include “pandemic crisis,” “state of alarm,” “emergency intervention,” “shortage,” “war economy,” and “health care collapse,” thus renewing, once again, the old relationship between crisis experiences and sociology, which gave rise to the discipline itself (Ramos and Callejo 2016).

2 Political discourse was filled with expressions such as “the Ifema miracle” or the “miracle hospital” (Community of Madrid 2020a).

3 This hypothesis became more evident a year later and was echoed by some headlines in leading Spanish media—e.g., “The Ifema hospital took away the medical staff who were going to medicalize the nursing homes” (Peinado 2021); or “[Former general director of Social and Health Coordination in Madrid] Carlos Mur acknowledges that there were protocols that indicated not referring elderly people from nursing homes to hospitals” (Telemadrid 2021).

4 According to Monica Greco (2004), this generic consensus is crystallized in the right to health, a form of protection that became generalized throughout the construction of the modern state not as an absolute right—as health is already too ambiguous a term and thus cannot be guaranteed under any circumstances—but as the possibility of accessing certain means and care that can potentially guarantee health.

5 News headlines such as “The Palacio de Hielo, Spain's great morgue, is the very image of the pandemic” (Latorre 2020) or “Palacio de Hielo morgue closes after holding the bodies of 1,146 people who died from COVID‐19” (Europa Press 2020) show how grim the situation we are describing was. While this temporary facility was in operation, access by photojournalists was carefully controlled to prevent potentially sensationalist coverage of what was already a very surreal phenomenon. For that reason, there are only a few snapshots of the facility, taken while it was being set up and when it was dismantled.

6 These are Orders SND/275/2020 and SND/352/2020, respectively.

7 The documentation we used for this analysis has not been published. Following a formal request and after months of waiting for a response, the Community of Madrid provided the disaggregated tables with data as of April 1, 2020, the peak of the first wave in this region. These do not show all the information requested, but they do include the main categories of interest to us, namely: deaths in nursing homes; deaths in hospitals; and deaths of patients with a COVID‐19 diagnosis and compatible symptomatology.
